# Comparative Expression of Renin-Angiotensin Pathway Proteins in Visceral Versus Subcutaneous Fat

**DOI:** 10.3389/fphys.2018.01370

**Published:** 2018-10-10

**Authors:** Yuebo Zhang, Kiran R. Somers, Christiane Becari, Katarzyna Polonis, Michaela A. Pfeifer, Alina M. Allen, Todd A. Kellogg, Naima Covassin, Prachi Singh

**Affiliations:** ^1^Department of Cardiovascular Medicine, Mayo Clinic, Rochester, MN, United States; ^2^Division of Gastroenterology and Hepatology, Mayo Clinic, Rochester, MN, United States; ^3^Department of Surgery, Mayo Clinic, Rochester, MN, United States

**Keywords:** obesity, fat distribution, visceral fat, renin-angiotensin system, angiotensin, chymase, Mas receptor

## Abstract

Body fat distribution contributes to obesity-related metabolic and cardiovascular disorders. Visceral fat is more detrimental than subcutaneous fat. However, the mechanisms underlying visceral fat-mediated cardiometabolic dysregulation are not completely understood. Localized increases in expression of the renin angiotensin system (RAS) in adipose tissue (AT) may be implicated. We therefore investigated mRNA and protein expression of RAS components in visceral versus subcutaneous AT using paired samples from individuals undergoing surgery (*N* = 20, body mass index: 45.6 ± 6.2 kg/m^2^, and age: 44.6 ± 9.1 years). We also examined RAS-related proteins in AT obtained from individuals on renin angiotensin aldosterone system (RAAS) targeted drugs (*N* = 10, body mass index: 47.2 ± 9.3 kg/m^2^, and age: 53.3 ± 10.1 years). Comparison of protein expression between subcutaneous and visceral AT samples showed an increase in renin (*p* = 0.004) and no change in angiotensinogen (*p* = 0.987) expression in visceral AT. Among proteins involved in angiotensin peptide generation, angiotensin converting enzyme (*p* = 0.02) was increased in subcutaneous AT while chymase (*p* = 0.001) and angiotensin converting enzyme-2 (*p* = 0.001) were elevated in visceral fat. Furthermore, visceral fat expression of angiotensin II type-2 receptor (*p* = 0.007) and angiotensin II type-1 receptor (*p* = 0.031) was higher, and MAS receptor (*p* < 0.001) was lower. Phosphorylated-p53 (*p* = 0.147), AT fibrosis (*p* = 0.138) and average adipocyte size (*p* = 0.846) were similar in the two depots. Nonetheless, visceral AT showed increased mRNA expression of inflammatory (TNFα, *p* < 0.001; IL-6, *p* = 0.001) and oxidative stress markers (NOX2, *p* = 0.038; NOX4, *p* < 0.001). Of note, mRNA and protein expression of RAS components did not differ between subjects taking or not taking RAAS related drugs. In summary, several RAS related proteins are differentially expressed in subcutaneous versus visceral AT. This differential expression may not alter AngII but likely increases Ang1-7 generation in visceral fat. These potential differences in active angiotensin peptides and receptor expression in the two depots suggest that localized RAS may not be involved in differences in visceral vs subcutaneous AT function in obese individuals. Our findings do not support a role for localized RAS differences in visceral fat-mediated development of cardiovascular and metabolic pathology.

## Introduction

Obesity results in heightened vulnerability to cardiovascular and metabolic disorders (Saydah et al., 2014). Among the obesity-related factors which contribute to adverse cardiometabolic effects, accumulation of visceral fat is critical. The role of visceral fat in cardiovascular and metabolic disease is suggested by cross-sectional studies and supported by experimental studies of weight gain (Fox et al., 2007; Orr et al., 2008; Mahabadi et al., 2009; Parikh et al., 2009; Liu et al., 2010; Romero-Corral et al., 2010; Chandra et al., 2014; Covassin et al., 2018). However, the molecular mechanisms mediating the pathological effects of visceral fat are not completely understood (Tchernof and Despres, 2013).

The renin angiotensin system (RAS) is a systemic hormone system which regulates blood pressure and plays a role in energy homeostasis (Marcus et al., 2013; Littlejohn and Grobe, 2015; Chappell, 2016). RAS constitutes a complex cascade of pathways through which AGT is converted to form bioactive peptides, AngII and angiotensin 1–7 (Ang1-7), via multistep-enzymatic processes including renin, ACE, chymase, and ACE homolog-2 (ACE2) (Chappell, 2016). These peptides mediate their cellular effects through AngII type-1 receptor (AT1R), AngII type-2 receptor (AT2R), and MASR. Of note, the AngII and Ang1-7 peptides have effects which are antagonistic to each other. AngII is proinflammatory, profibrotic and has vasoconstrictive effects, while Ang1-7 is anti-inflammatory, anti-fibrotic and has vasodilatory effects (Simoes e Silva et al., 2013). Similar contrasting effects of AngII peptide are also evident in cells based on activation of AT1R versus AT2R (Akazawa et al., 2013). Among the cellular signaling pathways, p53 plays a prominent role in RAS. Activation of p53 increases AGT and AT1R expression (Leri et al., 1998, 2000; Fiordaliso et al., 2001). Conversely, AngII activates p53 pathway to mediate its downstream cellular effects (Grishko et al., 2003; Liu et al., 2009; Guan et al., 2013). Furthermore, p53 has been shown to play an important role in oxidative stress, insulin signaling, angiogenesis, apoptosis, inflammation, and fibrosis in different cells including AT (Minamino et al., 2009; Shimizu et al., 2012; Ghosh et al., 2013; Gogiraju et al., 2015).

Obesity is associated with increased systemic RAS activation (Sarzani et al., 2008). Several animal studies demonstrate increases in RAS with high fat feeding and development of metabolic disorders (Favre et al., 2015; Littlejohn and Grobe, 2015). Conversely, blocking RAS via targeting AT1R, ACE1 activity, and/or (pro) renin receptor induces weight loss along with decreases in visceral obesity (Lee et al., 2008; Mathai et al., 2008; de Kloet et al., 2009; Favre et al., 2015; Littlejohn and Grobe, 2015; Tan et al., 2016; Azushima et al., 2017). Indeed, weight loss in humans is also associated with reduced RAS activity (Engeli et al., 2005; Wang et al., 2012). However, use of RAS targeted drugs has been largely shown to have no effect on body weight in clinical trials (Nedogoda et al., 2013; Littlejohn and Grobe, 2015). Of note, the role of AT1R in obesity-related pathology is evident in studies showing that RAS inhibitors improve glucose metabolism and reduce diabetes incidence in patients with metabolic syndrome (Investigators et al., 2006; Group et al., 2010). Among the factors contributing to increased RAS in obesity, the paracrine contribution of AT to systemic RAS has been suggested (Sarzani et al., 2008; Marcus et al., 2013). Several RAS components are present in AT where they locally regulate the micro-environment and thereby alter AT function (Sarzani et al., 2008; Marcus et al., 2013). AngII activation of the AT1R causes increases in adipocyte size, AT inflammation, oxidative stress, fibrosis, and decreases insulin sensitivity (Furuhashi et al., 2004; Azushima et al., 2017) while AngII activation of the AT2R can cause browning of the white AT, and decreases inflammation (Than et al., 2017). Similarly, increasing circulating Ang1-7 has been also shown to improve glucose and lipid metabolism, attenuate AT inflammation induced by a high fat diet, and also decreases abdominal fat mass (Santos et al., 2010, 2012; Liu et al., 2012; Schuchard et al., 2015).

Considering the varied and opposing roles of RAS in AT, differential expression of RAS components in regional fat depots may underlie the pathological effects of visceral obesity (Marcus et al., 2013; Littlejohn and Grobe, 2015). Hence we first examined the expression of RAS components in paired abdominal subcutaneous versus visceral AT. We hypothesized that compared to subcutaneous AT, visceral fat will have increased expression of RAS proteins involved in the pro-fibrotic AngII/AT1R pathway but attenuated expression of proteins mediating the anti-fibrotic Ang1-7/MASR pathway. We also determined the activation of the p53 signaling pathway, fibrosis, inflammation, oxidative stress, and adipocyte size in the subcutaneous versus visceral fat depots to evaluate potential detrimental downstream effects of RAS activation. Since RAAS targeted drugs are therapeutically used for hypertension, we next evaluated the effects of RAAS-targeted drugs on AT expression of RAS-related proteins in subcutaneous and visceral fat. We hypothesized that use of RAAS-targeted drugs will attenuate the expression of proteins associated with detrimental effects of RAS in AT.

## Materials and Methods

### Human Subjects

Adipose tissue samples obtained from 20 subjects (19 females) undergoing bariatric surgery were used to test our hypothesis. Pre-surgery clinical records were reviewed to obtain demographics and clinical information. Presence of diabetes, hypertension, and dyslipidemia was identified by clinical notes or medications. Sleep apnea was identified by overnight oximetry, polysomnography or clinical notes related to sleep apnea treatment. Additional AT samples from 10 subjects (6 females) undergoing bariatric surgery and prescribed RAAS targeted drugs were examined for depot specific alterations in AT associated with RAAS targeted therapy. Of these 10 subjects, 4 subjects were prescribed an ACE inhibitor (lisinopril), 4 subjects were taking an angiotensin receptor blocker (losartan), and 2 subjects were prescribed an aldosterone receptor antagonist (spironolactone). Demographics of the study population are detailed in **Table 1**. The study was approved by the Institutional Review Board and written informed consent was obtained from all subjects.

**Table 1:** Characteristics of the study subjects.

	No prescription of RAAS related drugs (*N* = 20)	Prescribed RAAS targeted drugs (*N* = 10)
Age [years]	44.6 ± 9.1	53.3 ± 10.1^∗^
Weight [kg]	126.5 ± 22.8	132.8 ± 37.8
Body Mass Index [kg/m^2^]	45.6 ± 6.2	47.2 ± 9.3
Diabetes, *n* [%]	7 [35%]	6 [60%]
Hypertension, *n* [%]	10 [50%]	10 [100%]^#^
Dyslipidemia, *n* [%]	12 [60%]	5 [50%]
Sleep Apnea, *n* [%]	14 [70%]	9 [90%]

*For continuous variables, data presented as mean ± SD. ^∗^P < 0.05 as determined by Wilcoxon Rank Sums Test. ^#^P < 0.05 as determined by 2-tailed Fisher’s Exact Test.*

Visceral (omental) and subcutaneous fat samples were endoscopically obtained during surgery. Biopsy samples were immediately brought to the lab, aliquoted for mRNA analysis, Western blot analysis and paraffin sectioning, and stored at -80°C for batched analysis.

### mRNA Analysis

Transcription of RAS components, inflammation and oxidative stress markers in the two fat depots was determined by standard reverse-transcription PCR using commercially available TaqMan probes (Applied Biosystems, Foster City, CA, United States). Briefly, AT was homogenized in Trizol reagent (Life Technologies, Carlsbad, CA, United States) and centrifuged to remove lipid layer. RNA containing aqueous phase was collected after treatment with chloroform. Total RNA from the aqueous phase was precipitated using ethanol and purified using PureLink RNA isolation kit (Life Technologies, Carlsbad, CA, United States). cDNA library was created using high-capacity cDNA reverse transcription kit (Applied Biosystems) for semi-quantitative determination of specific mRNA using the following commercial TaqMan probes per manufacturer instructions: AGT (Hs01586213_m1), renin (Hs00982555_m1), ACE (Hs00174179_m1), chymase (Hs01095979_g1), ACE2 (Hs01085333_m1), AT1R (Hs00258938_m1), AT2R (Hs02621316_s1), MASR (Hs00267157_s1), pro-renin receptor (Hs00997145_m1), p53 (Hs01034249_m1), IL-6 (Hs00174131_m1), TNFα (Hs00174131_m1), NOX2 (Hs00166163_m1), NOX4 (Hs01379108_m1), and GAPDH (Hs02786624_g1). Data were analyzed by comparative CT method using GAPDH as endogenous control (Schmittgen and Livak, 2008).

### Western Blot Analysis

The expression of RAS related proteins in the two depots was determined by standard Western blot analysis. Briefly, AT was pulverized in liquid nitrogen, suspended in RIPA buffer (Thermo Fisher Scientific Inc., Waltham, MA, United States) containing protease inhibitor cocktail (Millipore Sigma, Burlington, MA, United States) followed by centrifugation to remove lipid layer and insoluble pellet. Equal amount of protein from each sample was separated by PAGE and transferred onto PVDF membrane. The membrane were blocked with 5% non-fat milk and incubated overnight at 4°C with specific primary antibody for chymase (sc-59589, Santa Cruz Biotechnology, Dallas, TX, United States), AGT (origene-CF804670, OriGene Technologies Inc., Rockville, MD, United States), renin (sc-22752), ACE (ab28311, Abcam, Cambridge, MA, United States), ACE2 (MABN59, Millipore Sigma), AT2R (sc-9040), AT1R (ab124734), MASR (sc-54848, phospho-p53 (ab1431), p53 (9282s, Cell Signaling Technology, Danvers, MA, United States), and GAPDH (endogenous control, ab8245). After incubation with specific primary antibody, the membrane was washed and incubated with appropriate secondary antibodies, washed and developed using Luminata Forte Western HRP substrate (WBLUF0100, Millipore Sigma). Images were acquired with Odyssey Fc Image System (LI-COR Corporate, Lincoln, NE, United States) and analyzed using LI-COR Image studio (version 4.0). Protein expression was normalized to loading control (GAPDH). Abdominal subcutaneous and visceral fat samples from each subject were run on the same gel to minimize variations.

### Measurement of Fibrosis and Adipocyte Size

Adipose tissue samples were paraffin embedded, sectioned and stained for fibrosis using Masson’s trichrome staining at the Mayo Clinic core facility (Arizona) following standard protocols. Quantitation of fibrosis was done using Image J software using standard instructions^1^. For each tissue sample, percent fibrosis was determined in 5 random images and average value was used for further analysis. Furthermore, adipocyte size was determined using Aperio Image Scope software (Leica Biosystems, Buffalo grove, IL, United States) in 8 random areas per sample.

### Statistical Analysis

Data distribution was examined. Normally distributed data are reported as mean ± SD. Non-normally distributed data are reported as median along with interquartile range. The differences between the abdominal subcutaneous and visceral AT, and drug usage were assessed using repeated ANOVA. Prior to the analysis, skewed variables were log transformed to approximate normal distribution. All analyses were performed using JMP Pro 13.0.0 and *P* < 0.05 was considered statistically significant.

## Results

### Angiotensin Peptide Generation Pathways

**FIGURE 1:**
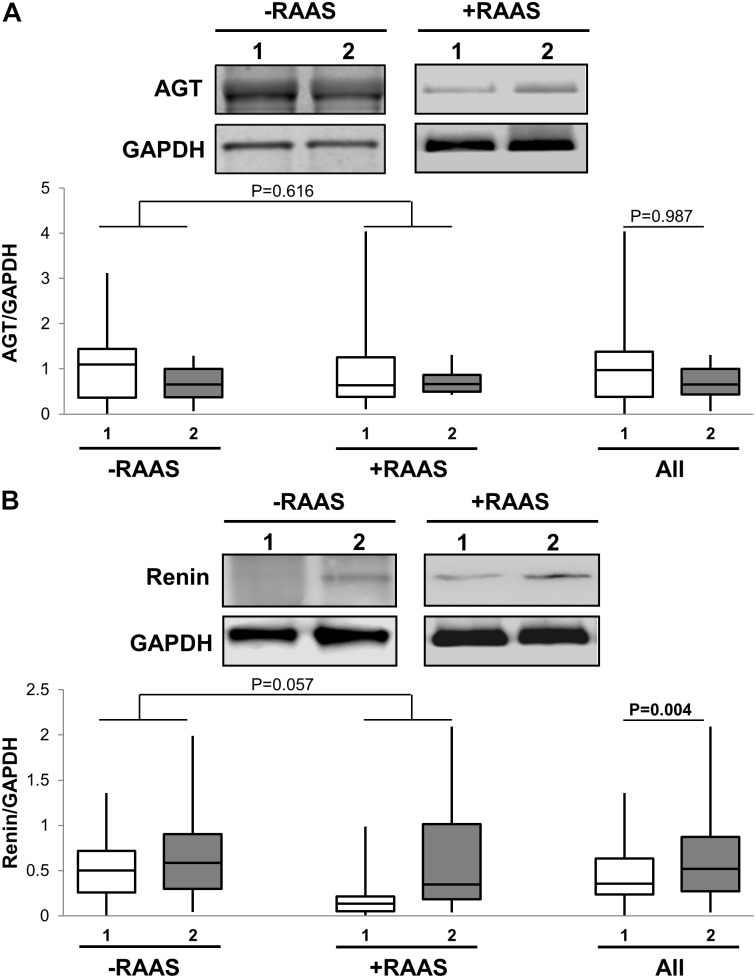
Angiotensin I Generation Pathway. Representative Western blots and graphs showing expression of AGT, **(A)** and renin **(B)** in abdominal subcutaneous (**1**, white bars) and visceral (**2**, gray bars) fat depots. Data are presented as median and interquartile range. Fiskars depict minimum and maximum values. –RAAS: data from AT of subjects not taking any RAAS related drugs; +RAAS: data from AT of individuals taking RAAS targeted drugs. *P*-values were determined using repeated ANOVA.

The first step in generation of bioactive angiotensin peptides involves conversion of inactive AGT to AngI by enzymatic renin activity. Therefore, we examined the mRNA and protein expression of both AGT and renin in subcutaneous versus visceral fat depots. Increased transcription of AGT in visceral fat was evident (**Table 2**). However, mRNA for renin was not detected in most study samples. At the protein level, we showed that AGT expression was not different between the two depots while renin expression was increased in visceral fat (**Figure 1**). Additionally, the mRNA and protein expression of AGT and renin did not vary with use of RAAS targeted drug and there were no interactions between drug use and depots (protein expression: AGT *p* = 0.959; renin *p* = 0.162).

**Table 2:** Transcription of RAS components in AT.

mRNA ratio	-RAAS (*n* = 20)		+RAAS (*n* = 10)		*P*-Value		
	Subcutaneous	Visceral	Subcutaneous	Visceral	Depot	Drug	Depot^∗^Drug
AGT/GAPDH	0.0059 (0.0122-0.0025)	0.0286 (0.0391-0.0162)	0.0046 (0.0107-0.0032)	0.0226 (0.0354-0.0134)	**<0.001**	0.55	0.7817
RENIN/GAPDH	ND	ND	ND	ND	-	-	-
ACE/GAPDH	0.0056 (0.0129-0.0045)	0.0287 (0.0452-0.0168)	0.0062 (0.0223-0.0029)	0.024 (0.0415-0.0116)	**<0.001**	0.887	0.661
Chymase/GAPDH	0.0051 (0.0076-0.0015)	0.0106 (0.0191-0.0052)	0.0036 (0.0148-0.0004)	0.0076 (0.0135-0.0057)	**0.001**	0.522	0.614
ACE2/GAPDH	0.0002 (0.0005-0.0002)	0.0016 (0.0034-0.0011)	0.0002 (0.0013-0.0001)	0.0028 (0.004-0.0015)	**<0.001**	0.242	0.93
AT1R/GAPDH	0.0011 (0.0017-0.0004)	0.0017 (0.0032-0.001)	0.0015 (0.0017-0.0012)	0.002 (0.0025-0.0011)	**0.03**	0.595	0.269
AT2R/GAPDH	0.0003 (0.0008-0.0002)	0.001 (0.0013-0.0007)	0.0002 (0.0008-0.0002)	0.0012 (0.0025-0.0005)	**<0.001**	0.956	0.265
MASR/GAPDH	0.0005 (0.0012-0.0002)	0.0009 (0.0014-0.0003)	0.0004 (0.0011-0.0001)	0.0013 (0.0067-0.0004)	**0.011**	0.786	0.058
P53/GAPDH	0.0036 (0.0454-0.0015)	0.0456 (0.0673-0.034)	0.0039 (0.0785-0.0015)	0.0607 (0.0786-0.0318)	**<0.001**	0.642	0.6613
Pro-renin receptor/GAPDH	0.1402 (0.1838-0.0339)	0.0874 (0.1518-0.0533)	0.1283 (0.1926-0.061)	0.0589 (0.1311-0.0401)	0.498	0.343	0.735

*Data presented as median and interquartile range. –RAAS refers to measures obtained from AT of subjects not taking any RAAS related drugs. +RAAS refers to measures obtained from AT of subjects prescribed RAAS targeted drugs. P-values were determined using repeated ANOVA. ND: not detected. Significant *P*-values are bolded.*

**FIGURE 2:**
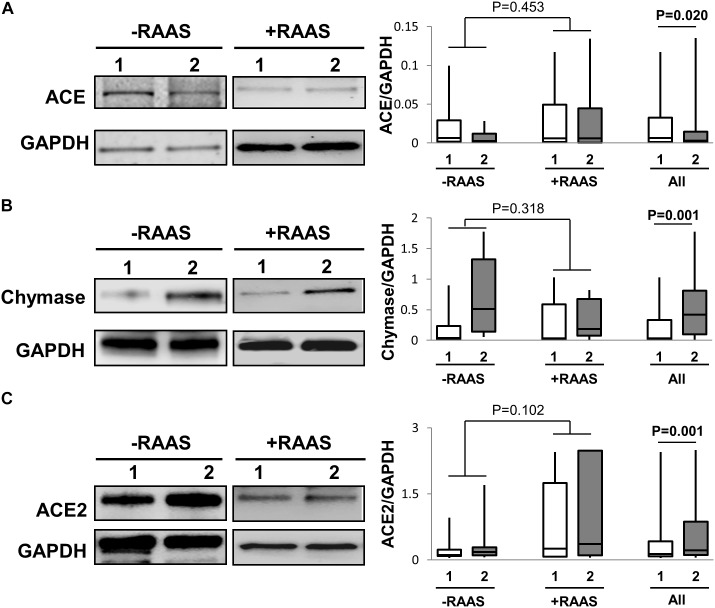
Pathways to AngII and Ang1-7 Peptide Generation. Representative Western blots and graphs showing expression of ACE, **(A)**, chymase **(B)** and ACE2, **(C)** in abdominal subcutaneous (**1**, white bars) and visceral (**2**, gray bars) fat depots. Data are presented as median and interquartile range. Fiskars depict minimum and maximum values. –RAAS: data from AT of subjects not taking any RAAS related drugs; +RAAS: data from AT of individuals taking RAAS targeted drugs. *P*-values were determined using repeated ANOVA.

Further, the predominant classical RAS pathway involves hydrolysis of AngI to AngII by the metallopeptidase ACE. However, the conversion AngI to AngII is also enabled by the enzyme chymase. Hence, we determined the expression of both ACE and chymase. While ACE protein expression was lower, chymase protein expression was higher in visceral fat (**Figure 2**). Unlike protein expression, increased mRNA for both ACE and chymase were seen in visceral AT (**Table 2**). The alternate counter-regulatory RAS axis (Ang1-7/MASR) requires ACE2 activity to generate Ang1-7. The expression of ACE2 protein (**Figure 2**) and mRNA (**Table 2**) was elevated in visceral fat. The mRNA and protein expression of ACE, chymase, and ACE2 did not vary with use of RAAS targeted drugs and no interaction between depots and drug usage was apparent (protein expression: ACE *p* = 0.500; chymase *p* = 0.204; ACE2 *p* = 0.867).

### Cellular RAS Receptors

**FIGURE 3:**
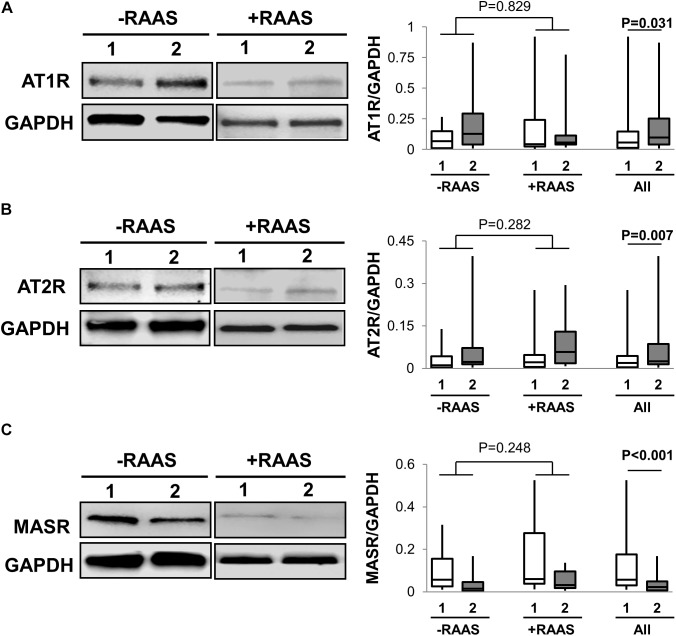
Cellular Receptors for Angiotensin Peptides. Representative Western blots and graphs showing expression of AT1R, **(A)**, AT2R, **(B)** and MASR in **(C)** abdominal subcutaneous (**1**, white bars) and visceral (**2**, gray bars) fat depots. Data are presented as median and interquartile range. Fiskars depict minimum and maximum values. –RAAS: data from AT of subjects not taking any RAAS related drugs; +RAAS: data from AT of individuals taking RAAS targeted drugs. *P*-values were determined using repeated ANOVA.

Classical RAS mediates its contrasting cellular action through mainly AT1R and AT2R while the alternate RAS axis requires MASR for its downstream cellular effects. The mRNA and protein expression of AT1R and AT2R was increased in visceral fat (**Table 2** and **Figure 3**). However, MASR mRNA expression was higher while MASR protein expression was lower in visceral AT. We also showed that the transcription of pro-renin receptor was not different between the two depots (**Table 2**). Similar to proteins involved in angiotensin peptide generation, the mRNA and protein expression of cellular RAS receptors did not show any alterations related to RAAS targeted drug use or interactions of drug use with depots (protein expression: AT1R *p* = 0.187; AT2R *p* = 0.57; MASR *p* = 0.198).

### Adipose Tissue p53, Fibrosis, Adipocyte Size, Oxidative Stress, and Inflammation

**FIGURE 4:**
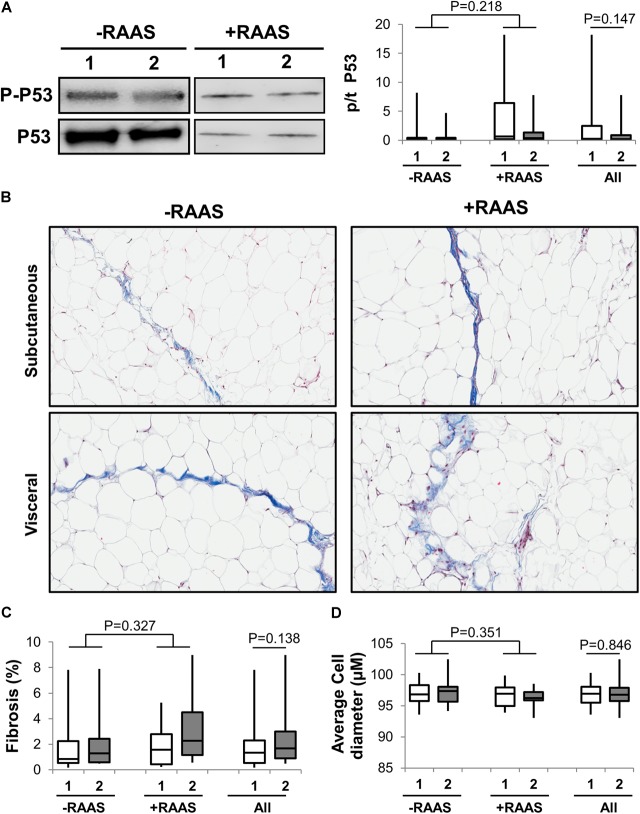
Adipose Tissue p53 Activation, Fibrosis and Cell Size. Representative Western blots and graph showing expression of phosphorylated p-53 **(A)** in abdominal subcutaneous (**1**, white bars) and visceral (**2**, gray bars) fat depots. Representative trichrome stained images **(B)** and graphs quantifying fibrosis **(C)** and average adipocyte size **(D)** from subcutaneous and visceral fat depots. Data are presented as median and interquartile range. Fiskars depict minimum and maximum values. –RAAS: data from AT of subjects not taking any RAAS related drugs. +RAAS:data from AT of individuals taking RAAS targeted drugs. *P*-values were determined using repeated ANOVA.

**FIGURE 5:**
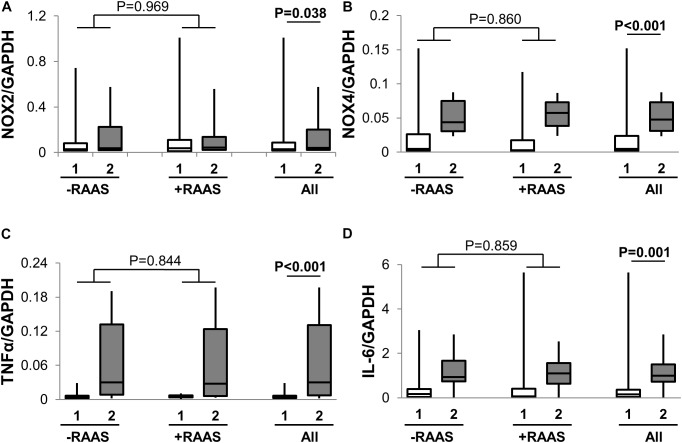
Adipose Tissue Oxidative Stress and Inflammation. Graph showing transcription of NOX2 **(A)**, NOX4 **(B)**, TNFα **(C)**, and IL-6 **(D)** in abdominal subcutaneous (**1**, white bars) and visceral (**2**, gray bars) fat depots. Data are presented as median and interquartile range. Fiskars depict minimum and maximum values. –RAAS: data from AT of subjects not taking any RAAS related drugs. +RAAS: data from AT of individuals taking RAAS targeted drugs. *P*-values were determined using repeated ANOVA.

Considering that several of the detrimental cellular effects of AngII are mediated via activation of the p53 pathway, we examined the expression of phosphorylated and total p53 in visceral and subcutaneous fat samples. Phosphorylated p53 and total p53 did not differ between the two fat depots (**Figure 4**). However, increased transcription of p53 mRNA was observed in visceral fat (**Table 2**). Next, we quantified fibrosis in visceral and subcutaneous AT as it is considered a surrogate marker for AT dysfunction and a downstream detrimental effect of RAS (Sun et al., 2013). Tissue fibrosis was similar in the two fat depots in our study subjects irrespective of RAAS drug usage and no interactions between depots and drug use was seen (*p* = 0.606, **Figure 4**). To gain further insights, we also examined average adipocyte size along with mRNA expression of inflammatory (IL-6 and TNFα) and oxidative stress markers (NOX2 and NOX4). Adipocyte size was not different in the two fat depots (**Figure 4**) and was not altered by RAAS drug usage. However, increased transcription of IL-6 and TNFα was observed in visceral fat along with increases in NOX2 and NOX4 mRNA (**Figure 5**). Also, no interactions with use of RAAS targeted drug were seen (IL-6 *p* = 0.783; TNFα *p* = 0.491; NOX4 *p* = 0.594; NOX2 *p* = 0.570)

## Discussion

The main finding of our study is that even though several components of the renin-angiotensin pathway are differentially expressed in the subcutaneous and visceral fat depots, the overall effects of these alterations do not appear likely to contribute to increased RAS activity in visceral fat. This is contradictory to our hypothesis that visceral fat would have increased expression of AngII/AT1R but decreased expression of Ang1-7/MASR and AT2R. This is the first study, in humans, to examine the expression of RAS related proteins and mRNA including both the traditional as well as the alternate RAS axis in paired visceral and subcutaneous AT. Previous studies only examined the transcription of the proteins comprising the traditional RAS axis (AGT/AngII/AT1R) (Dusserre et al., 2000; Giacchetti et al., 2000, 2002).

Renin angiotensin system is a complex system with multiple functional arms which may counterbalance each other. AngI generation is the first step toward formation of bioactive angiotensin peptides. On the one hand, we observed an increased transcription of AGT and renin in visceral AT. On the other hand we show that AGT protein expression was similar in the two fat depots but renin protein expression was increased in visceral fat. Nonetheless, the increases in renin protein expression in visceral fat may suggest a differential increase in AngI peptide in visceral fat depots. However, conclusive evidence can be only drawn from measuring renin activity which was beyond the scope of this manuscript. Our mRNA findings are consistent with the previous studies showing increased AGT mRNA expression in visceral fat (Dusserre et al., 2000; Giacchetti et al., 2000, 2002). The difference in AGT mRNA and protein expression may result from secretion of AGT peptide thereby resulting in no change in intracellular AGT protein levels. The discrepancies in mRNA and protein data related to AGT expression could also suggest post-transcription/translational mechanisms which may reduce stability of AGT protein expression and requires further investigation. Similar inconsistencies between mRNA and protein data were also observed for ACE, MASR and p53.

Among enzymes involved in AngII generation, we show that while ACE protein expression is higher in subcutaneous AT, chymase protein expression is higher in visceral fat. Hence even though differences in ACE and chymase protein expression exist, these differences may be redundant and lead to similar levels of AngII in the two fat depots. Nevertheless, our data suggest that since AngII generation in visceral fat may be more dependent on chymase activity, the beneficial therapeutic effects of ACE inhibitors in reducing AngII levels in visceral fat may be less effective. Of note, overall expression of RAS related proteins in AT of individuals taking and not taking RAAS directed therapeutics was similar, and no interaction between depot differences and drug use was observed. Several beneficial effects of RAS are mediated through the alternate axis comprising Ang1-7/MASR. Ang1-7 generation is dependent on the enzymatic actions of ACE2. Importantly, ACE2 enzymatic activity serves a dual purpose. First, it reduces the availability of AngII, and second, it forms Ang1-7. We demonstrate that ACE2 expression is higher in visceral AT, which suggests that Ang1-7 generation may also be higher and this could also likely lower AngII in visceral fat. These findings highlight the need for future studies examining angiotensin peptides in regional fat depots.

Furthermore, we show that AT1R and AT2R have higher expression in visceral fat, and MASR has higher expression in subcutaneous fat. An increase in AT1R mRNA in visceral fat has been previously reported; (Giacchetti et al., 2002), however, expression of AT2R and MASR has not been examined before. The potential benefits of increases in Ang 1-7 generation (suggested by increased ACE2 expression) in visceral fat would likely be counteracted by decreases in MASR expression in visceral fat. It is also likely that the lower expression of MASR in visceral fat may be a reflection of increased Ang1-7 generation. Indeed, Ang1-7 is shown to cause internalization of MASR which may potentiate endosomal degradation (Gironacci et al., 2011).

The overall absence of RAS related differences in proteins involved in AngII generation along with decreases in MASR (possibly in conditions of increased Ang1-7) in visceral fat tissue is supported by similar levels of cellular p53 pathway activation, fibrosis, and adipocyte size. However, increased mRNA of proteins related to inflammation and oxidative stress in visceral fat was observed. Our findings are concordant with previous studies showing similar fibrosis between regional fat depots (Divoux et al., 2010; Muir et al., 2016). However, this is in contrast to a previous study which showed increased collagen deposition in visceral fat (Michaud et al., 2016). The lack of difference in AT fibrosis in the regional fat depots in our study may be related to our study population, which only includes obese individuals, and the method used to measure fibrosis. Importantly, previous studies, (Divoux et al., 2010; Michaud et al., 2016) reported a positive correlation with the amounts of fibrosis in omental and subcutaneous fat, suggesting that similar central mechanisms may be contributing to concomitant deposition of extracellular matrix in the two fat depots. This suggests that while increased RAS in obesity may still contribute to AT fibrosis, this may be related to central/systemic effects of RAS rather than localized regional differences in AT.

The strength of our study is in the comprehensive measurement of RAS including the classic as well as the alternate beneficial arm, at the mRNA and protein level. Our study also includes assessment of paired subcutaneous AT from individuals taking RAAS targeted drugs which provides insights into differential effects of these drugs on proteins of the RAS pathway in visceral versus subcutaneous tissue. Limitations include our study population which comprises mostly morbidly obese individuals with several comorbidities, and chronic usage of medications. Also, the lack of comparison with AT samples from normal-weight subjects limits our ability to comment on the impact of obesity on AT RAS. On the other hand, it is the morbidly obese population which is at greatest risk for visceral fat mediated cardiovascular and metabolic disease, and is the population of greatest interest regarding mechanisms through which visceral fat may elicit adverse consequences. Importantly, our population includes a large proportion of females which prevents generalization to males. Our study is also limited by the lack of availability of specific AT2R antibody (Hafko et al., 2013). To overcome this limitation, we have also provided mRNA data which, consistent with protein quantification, also show preferential increases in visceral AT.

In summary, even though there is differential expression of RAS proteins in subcutaneous versus visceral AT, these differences appear unlikely to contribute to changes in AT function and to cardiometabolic pathophysiology associated with visceral adiposity.

## Author Contributions

PS, YZ, KS, CB, AA, and TK conceptualized and designed the study. YZ, KS, CB, KP, and MP collected and analyzed the tissue samples. YZ and PS performed the data analysis. YZ, KS, CB, KP, MP, AA, TK, NC, and PS interpreted the data, drafted the manuscript, and approved the final manuscript.

## Conflict of Interest Statement

The authors declare that the research was conducted in the absence of any commercial or financial relationships that could be construed as a potential conflict of interest.

## Footnotes

1 https://imagej.nih.gov/ij/docs/examples/stained-sections/index.html

## Abbreviations

ACE: angiotensin converting enzyme
AGT: angiotensinogen
AngII: angiotensin II
Ang1-7: angiotensin 1-7
AT: adipose tissue
AT1R: angiotensin II type-1 receptor
AT2R: angiotensin II type-2 receptor
BMI: body mass index
MASR: Mas receptor
NOX: NADPH oxidase
RAS: renin-angiotensin system
RAAS: renin-angiotensin aldosterone system
